# Modulation of Milk Allergenicity by Baking Milk in Foods: A Proteomic Investigation

**DOI:** 10.3390/nu11071536

**Published:** 2019-07-06

**Authors:** Simona L. Bavaro, Elisabetta De Angelis, Simona Barni, Rosa Pilolli, Francesca Mori, Elio. M. Novembre, Linda Monaci

**Affiliations:** 1 Institute of Sciences of Food Production, Italian National Research Council (ISPA-CNR), Via Amendola 122/O, 70126 Bari, Italy; 2 Allergy Unit, Department of Pediatrics, Anna Meyer Children′s University Hospital, University of Florence, 50139 Florence, Italy

**Keywords:** milk allergen, baked milk, cow’s milk, allergenicity modulation, proteomics

## Abstract

Cow’s milk is considered the best wholesome supplement for children since it is highly enriched with micro and macro nutrients. Although the protein fraction is composed of more than 25 proteins, only a few of them are capable of triggering allergic reactions in sensitive consumers. The balance in protein composition plays an important role in the sensitization capacity of cow’s milk, and its modification can increase the immunological response in allergic patients. In particular, the heating treatments in the presence of a food matrix have demonstrated a decrease in the milk allergenicity and this has also proved to play a pivotal role in developing tolerance towards milk. In this paper we investigated the effect of thermal treatment like baking of cow’s milk proteins that were employed as ingredients in the preparation of muffins. A proteomic workflow was applied to the analysis of the protein bands highlighted along the SDS gel followed by western blot analyses with sera of milk allergic children in order to have deeper information on the impact of the heating on the epitopes and consequent IgE recognition. Our results show that incorporating milk in muffins might promote the formation of complex milk–food components and induce a modulation of the immunoreactivity towards milk allergens compared to milk baked in the oven at 180 °C for ten minutes. The interactions between milk proteins and food components during heating proved to play a role in the potential reduction of allergenicity as assessed by in vitro tests. This would help, in perspective, in designing strategies for improving milk tolerance in young patients affected from severe milk allergies.

## 1. Introduction

The introduction of cow’s milk (CM) in the human diet has been a very long tradition, for approximately 9000 years. Since then, the incidence of adverse reactions to CM is constantly increasing, becoming one of the first and most common causes of food allergies in early childhood in Europe [1]. The reported prevalence of cow’s milk allergy (CMA) varies considerably between studies probably due to the different methods used for diagnosis or the differences in the ages of the studied populations [2]. It has been reported that nowadays 0.6% to 3% of children under the age of 6 years, 0.3% of older children and teens, and less than 0.5% of adults suffer from CMA [3]. Although 15% of affected children remain allergic throughout adulthood, the majority of milk allergic infants seems to be able to consume milk and its by-products with a total resolution of CMA in 19% of the children by 4 years of age, in 42% by 8 years of age, in 64% by 12 years of age, and in 79% by 16 years of age. Despite these encouraging data, the mechanisms underpinning the development of clinical tolerance are not fully understood [4,5].

Cow’s milk contains approximately 30–35 g of proteins per liter encompassing more than 25 different proteins, although only some of them are capable of triggering allergic reactions. Proteins composing milk typically belong to two different categories: Caseins (αS1-casein, αS2-casein, β-casein and k-casein) and whey proteins (β-lactoglobulin [β-LG], α-lactalbumin [ALA], bovine lactoferrin [LF], bovine serum albumin [BSA] and bovine immunoglobulins [Ig]), accounting for respectively the 80% and 20% of the total cow milk protein content [6,7]. According to the World Health Organization and International Union of Immunological Societies (WHO/IUIS) official list of allergens, milk allergen proteins are classified with the following designation: Bos d 5 (β-LG), Bos d 4 (ALA), Bos d 6 (BSA), Bos d 7 (Ig), Bos d 9 (αS1-casein), Bos d 10 (αS2-casein), Bos d 11 (β-casein), Bos d 12 (κ-casein). From a biochemical point of view, caseins are phosphoproteins that exist as colloidal aggregates known as casein micelles, and whose function mainly consists in binding essential minerals, such as calcium phosphate, that would otherwise precipitate resulting difficult in being ingested [8]. In cow’s milk, four main casein phosphoproteins have been identified namely αS1-casein, αS2-casein, β-casein, and κ-casein, approximately in proportions respectively of 4:1:4:1 by weight. Their molecular weights range between 19 and 25 kDa, with an average isoelectric point (pI) comprised of between 4.6 and 4.8. Moreover, all caseins are amphiphilic and have well-defined structures, with a little primary structure homology while sharing biophysical features such as heat resistance [9]. On the contrary, whey proteins (WPs) consist mostly of β-LG (about 44.70%) and ALA (about 14.22%), along with Ig and BSA (about 1.5%) [10], with molecular weight between 14 and 80 kDa. Their structure is mainly composed of nine anti-parallel β-sheets and one α-helix, with two intra-molecular disulphide bonds and one free potentially reactive sulfhydryl group [11] that confers high stability against proteases and acidic hydrolysis [12].

The balance between these two different groups of protein plays an important role in the sensitization capacity of cow’s milk, and its modification can increase the immunological response in allergic patients [13]. However, the control of the daily intake of caseins/WPs ratio in allergic consumers is not easy, and the only useful action to protect them from developing adverse reactions due to CM consumption remains the strict avoidance of milk and dairy products. Nonetheless several strategies have been developed to promote safe consumption of milk and its derived products in allergic patients and efforts have been also directed to design the best procedures for inducing cow’s milk oral tolerance [14] or to calculate the safest dose to start from for oral desensitization studies [15]. Among these it has been demonstrated that the consumption of baked milk as such or as an ingredient included into the food matrix might induce milk tolerance in 50–70% of CM allergic children enrolled in the study [16,17]. This is also confirmed by other more recent works reporting that the consumption of baked goods containing egg or cow’s milk may hasten the development of tolerance to these foods in an unheated form [18,19,20]. This paves the way for setting up oral food challenge studies using milk-including baked goods to be administered to milk allergic patients.

The heating treatment and the matrix where the allergen is contained, play a pivotal role in developing tolerance towards milk. High temperature and prolonged cooking time, as well as intrinsic characteristics and physicochemical conditions of the cooking environment, can induce significant changes in proteins structure, such as destruction of conformational epitopes, alteration of allergens tridimensional structure, with a consequent decrease of the IgE-binding. It has been observed that heat treatments commonly applied during industrial processing could deeply affect milk protein stability. For instance, whey proteins tend to aggregate due to the interaction of a free–SH group with the S–S bond of cysteine-containing proteins, such as β-LG, κ-casein, ALA, and BSA via –SH/S–S interchange reactions [21]. In addition, an extensive interaction between matrix components and milk proteins could occur after heating, originating the so-called “matrix effect”, with consequent alteration of the final allergenicity. It has been hypothesized that the interaction between proteins and other components of the food matrix (fat or sugar) can alter protein structure and hide IgE binding sites. Schulten et al. demonstrated in 2011 that complex food matrices such as hazelnuts and peanuts can significantly reduce the gastrointestinal digestibility and the epithelial transport of cow’s milk and apple allergens, thereby reducing their final allergenicity [22].

In other investigations carried out in our group we demonstrated that application of thermal treatments can induce relevant changes in the protein structure hiding or destroying specific epitopes with promising results on the reduction of the allergenic potential [23,24].

According to the data obtained in this work, it can be speculated that all interactions leading to an irreversible aggregation of proteins into complexes of various molecular size as a consequence of heating and/or protein composition, can influence the allergic response. In support of this, several authors, studying the IgE- and IgG-binding affinity and stability of different allergic subjects, observed that the types and severity of reactions displayed in the same individual depended on the different physical and chemical modifications that proteins underwent upon food processing [17,25].

In this context, the aim of the current study is to widen, from an allergenic point of view, the knowledge about the effect of thermal treatment on cow’s milk proteins that were employed as ingredients in the preparation of muffins for infants. Any change in the protein profile was investigated by means of electrophoresis technique, and any possible protein–protein aggregation, polymerization or co-migration with the food matrix was highlighted. Furthermore, western blot analyses with sera of milk allergic children were performed in order to obtain deeper information on the impact of the applied heating on epitopes and consequent IgE recognition. This would help in better understanding the phenomena occurring along the protein structure and/or amino acid modification and their role in improving milk tolerance in young patients affected from severe milk allergies.

## 2. Materials and Methods

### 2.1. Chemicals

Trizma-base, sodium chloride, Tween-20, Triton X-100, ammonium bicarbonate (AMBIC), iodoacetamide (IAA), bovine serum albumin (BSA), along with other chemicals for electrophoresis namely dithiothreitol (DTT), sodium dodecyl sulfate (SDS), glycine, glycerol, Coomassie brilliant blue-G 250 were obtained from Sigma-Aldrich (Milan, Italy). Bromophenol blue was provided by Carlo Erba Reagents (Cornaredo, Italy) while phosphate buffer saline (PBS) was purchased from VWR International s.r.l. (Milan, Italy). Syringe filters in cellulose acetate (CA) from 1.2 µm were obtained from Labochem Science S.r.l. (Catania, Italy) whilst 0.45 µm syringe filters in polytetrafluoroethylene (PTFE) were purchased from Sartorius (Göttingem, Germania). Acetonitrile (Gold HPLC ultragradient), and trifluoroacetic acid (TFA) were purchased from Carlo Erba Reagents (Cornaredo, Milan, Italy) and ultrapure water was produced by a Millipore Milli-Q system (Millipore, Bedford, MA, USA). Formic acid (MS grade) was provided by Fluka (Milan, Italy) while trypsin (proteomic grade) for in gel protein digestion, from Promega (Milan, Italy).

### 2.2. Sera of Milk Allergic Patients

Sera were obtained from a total of 6 milk allergic children with levels of total IgE ranging from 203 to 5000 KU/L with an age comprised of between 5 and 16 years, according to ethical requirements. Tests were conducted in accordance with the Declaration of Helsinki and all procedures of the study were approved by the local Ethics Committee (code 2018/128). Permission to participate in the study of all children was obtained and the written informed consent was signed by the parents. The allergy symptoms in general ranged from urticaria to angioedema and anaphylaxis. The clinical features of the allergic individuals enrolled in this study are reported in Table 1. Diagnosis of IgE-mediated allergy to CM was confirmed by skin prick test (SPT) and serum-specific IgE (ImmunoCAP, Phadia, Uppsala, Sweden) to CM and CM proteins (s-IgE to CM, β-LG, ALA, caseins, total serum), allowing for a reliable diagnosis of IgE-mediated CMA. All of the serum sera samples were stored at −80 °C before their use.

### 2.3. Samples Preparation

Commercial fresh whole cow’s milk (submitted to High Temperature Short Time-HTST) used in the present study was purchased from a local store shortly after delivery. Muffins baked with cow’s milk, were prepared according to the following recipe: 60 g of wheat flour, 100 g of sugar, 1 sachet of vanillin, 8 g of baking powder, and 100 mL of fresh cow’s milk (approximately 0.85 g of milk proteins for each muffin). The muffin was baked in an oven for 30 min at 180 °C. Blank muffin samples were also produced by replacing milk with water (a total of 100 mL). In addition, in order to have additional information on the effects of heating on milk proteins stability/structural–chemical modifications, the same amount of milk used for muffin preparation (100 mL) was baked at 180 °C for 10 min in an oven in absence of the food matrix.

### 2.4. Protein Extraction and Quantification

Blank muffins and CM incurred muffins were coarsely ground by hand and submitted to protein extraction procedure along with pasteurized and baked liquid milk. Briefly, 1 part of ground muffins or milk was mixed with 2 parts of extraction buffer (PBS, pH 7.4 containing 1% of Tween 20 (*v/v*) and 0.4% of Triton X-100 (*v/v*)), homogenized for 35 s (5 cycles of 7 s each) in a blender (Sterilmixer 12 model 6805-50; PBI International) and then shaken overnight at room temperature in an orbital shaker (KS 4000 i-control shaker, IKA Works GmbH & Co. KG, Staufen, Germany). Afterwards, samples were centrifuged for 20 min at 12,000 g at 4 °C, the upper phase was discarded, and the supernatant was carefully collected and filtered through 1.2 µm CA syringe filters. Protein concentration of samples was calculated as mg/albumin equivalent by Bradford assay (Quick Start™ Bradford Protein Assay). Samples were stored at −20 °C until use and filtered through 0.45 µm PTFE filters just before electrophoretic analysis.

### 2.5. SDS-PAGE Analysis

Fifteen microgram of protein extracts from muffin and milk samples, were separated, under reducing conditions, by means of sodium dodecyl sulfate-polyacrylamide gel electrophoresis (SDS-PAGE) on an 8–16% polyacrylamide pre-cast gels (8.6 cm × 6.7 cm × 1 mm) using a Mini-Protean Tetra Cell equipment (Bio-rad Laboratories, Segrate, Milano, Italy). Samples were dissolved in a Laemmli buffer (62.5 mM TrisHCl, pH 6.8, 25% glycerol, 2% SDS, 0.01% Bromophenol Blue, 100 mM DTT) (1:1 ratio) and denatured for 5 min at 95 °C. As running buffer, a TGS (25 mM Tris, 192 mM Glycine, 0.1% SDS) solution was employed while electrophoretic separation was performed at 100 V. Gels were stained by using a Coomassie Brilliant Blue G-250 solution and the bands were detected on a ChemiDOC^TM^ MP Imaging system (Bio-Rad Laboratories, Segrate, Milano, Italy) and analyzed by using the software ImageLab 4.1. Precision Plus Protein^TM^ all blue standards (10–250 kDa, Bio-Rad Laboratories, Hercules, CA, USA) was used as protein molecular weight referencing.

### 2.6. In-Gel Protein Digestion

Selected protein bands were cut from the polyacrylamide gel and submitted to in gel-digestion procedure according to the protocol reported in our previous work [26]. Finally, each sample was resuspended in 70 µL of H_2_O/ACN, 90/10 + 0.1% formic acid (*v/v*) and 3 µL were further injected into LC/MS apparatus.

### 2.7. Protein Identification by Untargeted HR MS/MS Analysis

Protein bands were analyzed by using a Q-Exactive™ Plus Hybrid Quadrupole-Orbitrap™ Mass Spectrometer coupled to a Ultra-High-Performance Liquid Chromatography (UHPLC)pump systems (Thermo Fisher Scientific, San Josè, CA, USA). Peptides mixture was separated on an Acclaim^TM^ PepMap analytical column (1 mm × 15 cm × 3 µm, 100 Å porosity, Thermo Fisher Scientific) at a flow rate of 60 μL/min, using a binary gradient composed of H_2_O + 0.1% formic acid (solvent A) and CH_3_CN/H_2_O 80:20 + 0.1% formic acid (solvent B). The gradient elution program was as follows: 0–60 min linear from 10% to 60% B; quick increase to 80% B and isocratic for 10 min; then returning to 10% B and isocratic for 20 min for column re-conditioning. MS spectra were acquired in positive ion mode. The Heated Electrospray Ionization(HESI) ion source settings are reported here: Spray voltage at 3.4 kV, capillary temperature at 320 °C, sheath gas flow rate at 25 arbitrary units and S-lens at 55. The other MS settings are the same of what was reported in Bavaro et al. [24]. Raw data were processed via the commercial software Proteome Discoverer^TM^ version 2.1 (Thermo-Fisher-Scientific, San Josè, CA, USA) and protein identification was achieved by Sequest HT search against a milk customized database extracted by Uniprot DB basing on the taxonomy code of *Bos Taurus* (ID: 9913) and containing about 44,000 sequences. The identification of tryptic peptides produced by in gel digestion with trypsin was accomplished by setting at 5 ppm and 0.05 Da the mass tolerance on the precursor and fragment ions, respectively. Only trustful peptide–spectrum matches were accepted and in particular a minimum of three peptides or higher were the minimum criteria for protein identification by selecting a high confidence (FDR < 1%).

### 2.8. In Silico Analysis to Assess the Immunoreactivity of Milk Proteins after Baking Process

Peptide sequences identified from the excised and digested protein bands were finally screened by interrogating in the Immune Epitope Database (IEDB) database (https://www.iedb.org/) in order to detect epitope linear sequences involved in IgE immunoreactivity. The following filters were applied for IEDB screening: Linear sequence for epitope structure, exact match for Basic Local Alignment Search Tool (BLAST) option and human as host.

### 2.9. Immunoblot for IgE-Binding Assay

Six µg of proteins extracted from allergen free and allergen incurred muffins and pasteurized/ baked liquid milk were separated by electrophoresis under reducing conditions as already described in Section 2.5, and subsequently electroblotted on an immuno-blot low-fluorescence polyvinylidene fluoride (PVDF) membranes in 7 min (1.3 A, 25 V) using the Trans-Blot Turbo Transfer System (Bio-Rad Laboratories, Segrate, Milano, Italy).

Membranes were washed for 30 min (3 cycles of 10 min each) in TBS buffer containing 0.1% of Tween-20 (TBS-T) and then blocked for 2 h at room temperature with 3% BSA solution (prepared in TBS–T buffer). The membranes were incubated with pooled sera of a total of 7 young allergic patients previously diluted in TBS-T at 1/25 ratio and kept shaking overnight at 4 °C. After washing with TBS-T (3 cycles of 10 min each), membranes were incubated with monoclonal peroxidase-conjugated mouse anti-human IgE antibody (Sigma Aldrich, Milan, Italy) diluted in blocking solution (1/5000) and left shaking for 2 h at room temperature. Successively, membranes were washed with TBS-T (3 cycles of 10 min each) and then with TBS (30 min) before being incubated with Clarity chemiluminescence substrate (Bio-Rad Laboratories, Segrate, MI, Italy), 5 min prior to UV exposition. Images were acquired on a ChemiDoc^TM^ MP Imaging System.

## 3. Results and Discussion

### 3.1. Effects of Baking and Matrix on Milk Protein Profiles

**Figure 1:**
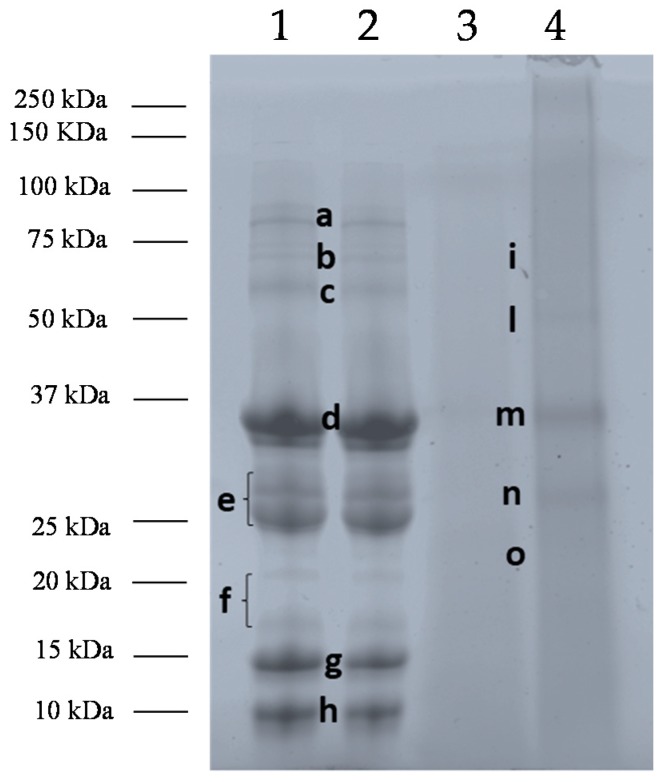
SDS-PAGE of cow’s milk (CM) submitted to the different treatments: Pasteurized CM (Lane 1), baked CM at 180 °C for 10 min (Lane 2), blank muffin (Lane 3) and CM incurred muffin (Lane 4).

At first, the stability of milk proteins submitted to baking treatment either as matrix-free liquid or included into a food matrix, such as a muffin, was investigated by SDS-PAGE analysis. After a deep inspection of the electrophoretic protein profiles, the most relevant bands were digested in-gel by trypsin and the resulting peptide pool subjected to discovery analysis by LC-HR-MS/MS platform. The MS spectra obtained for each individual band were processed via commercial software and the respective proteins identified by interrogating a refined *Bos Taurus* database available on-line from the UniProt portal. In Figure 1 the SDS-PAGE protein profiles of cow’s milk pasteurized (Lane 1), baked at 180 °C for 10 min (Lane 2), and blank muffin (allergen-free Lane 3), and CM incurred muffin, (Lane 4) were illustrated. Protein bands selected for identification were labeled from *a*–*h* (Lane 1) and *i-o* (Lane 4) (Figure 1). The results retrieved by the software for each spot analyzed were summarized in Table 2.

As displayed in Figure 1, no significant difference in the protein profile was observed between pasteurized and baked CM, suggesting that heating weakly affects the final stability of milk proteins in absence of the food matrix. In particular, protein bands with MW comprised between 75 and 50 kDa (Figure 1, Lanes 1 and 2, Band *c*), detectable in both extracts were mainly attributed to the whey protein BSA also named Bos d 6 that appeared as faint band in the first two lanes underlying the susceptible feature to the applied heating [27,28], while *a* and *b* did not lead to a univocal identification due to the low intensity of the protein bands.

Other bands uttermost intense displayed in pasteurized and baked milk were detected in the range of 37 and 25 kDa, namely *d* and *e* (Lane 1), and were assigned to caseins. Specifically, band *d* was attributed to αS1- and αS2 casein, (Bos d 9 and Bos d 10, respectively) while band *e* was identified as a mix of αS2-casein, β-casein (Bos d 11), and κ-casein (Bos d 12). Although clearly formed by two individual signals (see Figure 1, Lane 1, Band *e*), band *e* was experimentally processed as one and this could be the explanation for the different proteins identified. Our results highlight a good stability of caseins to heat treatments, as already reported in literature [7]. The heat resistance of the caseins group seems ascribable to a well-defined disordered mobile structure (rheomorphic) and to the lack of co-operative transition of unfolding, or partial folding, during heating [29]. Indeed, caseins lack a rigid tertiary structure, which confers stability and develop a “random coil” conformation stabilized by hydrophobic interactions [30]. Consequently, caseins are very stable to heat treatments, showing only a partial reduction or no change in their allergenicity [31]. Bloom and others demonstrated that the heat treatment after 60 min at 95 °C, did not affect the immunoreactivity of caseins [32]. Besides the time of heat exposure, the temperature or the presence of the food matrix (for example, wheat) during the heat process, casein allergenicity can be influenced also by digestion processes. Indeed, Morisawa et al. showed that α-caseins submitted to thermal treatment did not affect the histamine released from basophils, on the contrary, the combination of heat treatment with enzymatic digestion proved to decrease histamine released, reducing the interaction between α-casein specific-IgE and its linear epitopes [33]. In another study, Chatchatee et al. identified six major and three minor IgE epitopes of β-casein in persistent CMA patients. Among those, epitope 83–92 was the most frequently recognized (found in 13 out of 15 patients) and was identified by Dupont et al., in a tract highly resistant to digestion [34,35].

Other bands approximately comprised of between 20 and 10 kDa (Lanes 1, Bands *f*, *g* and *h*) were displayed in both pasteurized and baked milk protein profile (Figure 1, Lanes 1 and 2). By bioinformatic search, Band *f* was assigned to a mix of ALA (Bos d 4) and β-LG (Bos d 5), while bands *g* and *h* were singly attributed to β-LG and ALA respectively. Similarly to what already observed for other classes of proteins, baking seems not to affect β-LG and ALA stability. As reported in literature whey proteins are thermolabile with a consequent change in their allergenicity [36]. It is well known that β-LG increased its antigenicity and allergenicity when subjected to temperatures ranging from 50 to 90 °C, on the contrary a decrease was observed after 90 °C [37]. The fluctuated phenomena of β-LG thermal denaturation are characterized by well-defined temperature thresholds and lead to reversible and irreversible modifications [38,39]. Regarding ALA, several authors observed that this protein is more heat-stable than β-LG showing a greater decrease in antigenicity only when subjected to high temperatures, likely due to the loss of conformational epitopes that are more IgE-reactive [37,40]. It has been reported that the presence of fat and lactose enhanced β-LG denaturation in cow’s milk. Due to Maillard reaction, protein–lactose interactions occur during heating with consequent stabilization and increase of hydration of protein molecules and/or irreversible aggregation of the whey proteins with casein. Therefore, Maillard reaction may lead to a loss of β-LG linear epitopes and consequently reduces the antigenicity of the protein [32,37].

In Figure 1, protein profiles of blank muffin and milk incurred muffin extracts were also displayed (Lanes 3 and 4). At a glance, the electrophoretic pattern of milk proteins extracted from muffin (Lane 4) appears significantly different from those of pasteurized and baked milk. This different behavior is also due to the lower extraction yield of proteins processed and embedded into a complex food matrix. In particular, the bands markedly detectable in pasteurized and baked milk (Lanes 1 and 2, Bands *b, d,* and *e*) were visible in the milk containing muffin SDS-PAGE profile as weak signals (Lane 4, Bands *i, m,* and *n*), while bands *c*, *f*, *g,* and *h* of pasteurized milk (Lane 1) seems to disappear in the milk incurred muffin. Interestingly, a new band with MW comprised of between 20 and 25 kDa appeared in the milk muffin pattern (Band *o*, Lane 4). For identification purpose, bands *i-o* of the milk muffin profile were submitted to in-gel protein digestion, mass spectrometry analysis, and bioinformatic search. Specifically, band *i* was attributed to a mix of αS1- and β-casein, while bands *m* and *n* (Lane 4) were singly assigned to αS1- and β-casein, respectively. Additional band *o* (Lane 4) was identified as αS1-casein (see Table 2). As for blank muffin (Lane 3), no milk proteins were detected as expected. The differences observable between pasteurized/baked milk and CM incurred muffin protein profiles highlight well the importance of food matrix on protein stability to heating. In general, almost all classes of milk proteins (caseins and whey proteins) seems to be deeply affected by baking in the presence of the food matrix and this is confirmed by the reduction of signal intensity of the most intense bands in milk-incurred muffin or by the disappearance of specific bands. The only proteins detected in incurred muffin extract, although at a lower concentration, belong to casein (αS1- and ß-casein, bands *i*, *m*, *n*, *o*, Lane 4) which previously studies reported to be very stable to heat treatment. Whey proteins, such as BSA or ALA and ß-LG were not displayed in the gel. It should be hypothesized that the presence of the food matrix improved the heat dispersion within the environment where proteins are dispersed with a consequent increase of the temperature that induces protein degradation. On the other hand, it is well known that heating is likely to promote interaction between protein and other food components causing important structural and chemical changes in the proteins involved (denaturation, aggregation, and Maillard reaction) and altering the pH and the solubility and/or structure of allergenic proteins. In the light of this, it is reasonable to assume that the interaction of proteins with food components or alteration of their solubility might decrease the extraction efficiency preventing their detection in the final extract. In addition, the occurrence of some degradation phenomena during heating could explain the appearance of the new band at MW approximately below 22 kDa (Band *o*, Lane 4), putatively attributed to αS1- casein.. It was largely demonstrated that the interactions between milk proteins and matrix ingredients including proteins, fats, and sugars that are ingredients typically used in bakery products, can also decrease the bioavailability of allergic proteins to immune system and consequently reduce their allergenicity [41]. The interaction with matrix components has led some authors to recommend to young allergic patients to follow a diet including milk-containing bakery products like muffins. In these studies, about 70% of tested children were able to ingest a muffin containing baked milk without displaying any immediate clinical symptoms [17]. They suggest to add baked milk products into the daily diet in order to accelerate the rise of tolerance to unheated milk rather than to avoid strictly such allergenic food [20].

### 3.2. Immunoblot of Milk Products with Sera of Allergic Patients

**Figure 2:**
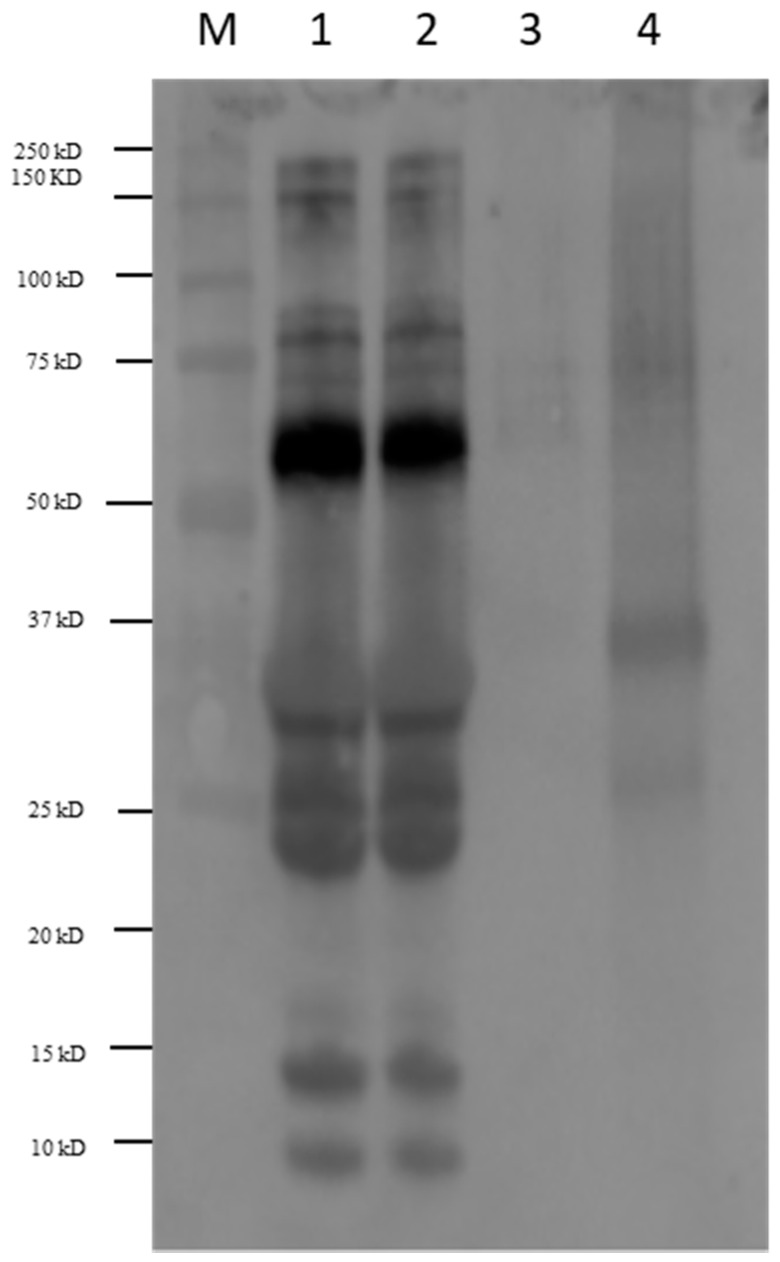
Immunoblot of CM sample extracts under reducing conditions referred to pasteurized CM (Lane 1), baked CM at 180 °C for 10 min (Lane 2), blank muffin (Lane 3), and CM incurred muffin (Lane 4). M: MW reference standard The immunoblot was carried out on a pool of sera of young patients (mean age ± standard deviation: 8.5 ± 4.3 years) with a clinical allergy to CM proteins.

In order to study the effect of baking in the final immunoreactivity of milk muffins, immunoblot analysis with sera of allergic young patients (mean age ± standard deviation: 8.5 ± 4.3 years) was performed. Specifically, pasteurized and baked milk along with muffins prepared with or without milk were separated on monodimensional electrophoresis, blotted on a PVDF membrane and detected by chemiluminescence reaction. A picture reporting the western blot analysis performed with pool sera of patients is shown in Figure 2. As appearing, pasteurized and baked milk showed a similar immunoreactivity profile (Lanes 1 and 2) suggesting that baking under these conditions did not alter the final allergenicity of that food. In particular, IgE immunoreactivity was detected in correspondence of bands comprised between 75 and 100 kDa, while a strong antibody reactivity was displayed for a band with MW of approximately 60 kDa (Figure 2, Lanes 1 and 2) experimentally assigned to BSA (Figure 1, Lane 1, Band *c*). The clinical relevance of BSA in milk is difficult to evaluate, as allergic children are generally sensitized to two or more milk allergen proteins [1], and BSA is a minor allergen [42]. Nevertheless, it was reported that 70% of patients with persistent milk allergy, have a greater risk to develop sensitization to bovine serum albumin (BSA). It was reported that heating treatment of milk, as boiling at 100 °C for 10 min, determines an increase of dimeric, trimeric and higher polymeric BSA forms, which maintain strong IgE-binding properties. Our results demonstrated that baking milk at 180 °C in the oven for 10 min did not significantly reduce BSA immunoreactivity, likely due to the ineffective spread of the temperature within milk during heating.

An IgE reactivity lower than the BSA band was instead observed in pasteurized and baked milk corresponding to bands with MW in the range 37–22 kDa (Figure 2 Lanes 1 and 2) namely bands labeled as *d* and *e* in the respective SDS-PAGE pattern (Figure 1, Lane 1) and assigned to the casein group (αS1/αS2-casein, β-casein and κ-casein). Our findings are in accordance with what was reported in literature. Indeed, as known among cow’s milk proteins, caseins showed a high heat stability with persistence of IgE-binding properties. Nevertheless, IgE binding depends on the clinical history of patients. Indeed, several authors observed that casein heated for a prolonged time produced consistent reactivity in some milk-reactive subjects, while reduced adverse reactions were observed in other milk-tolerant patients, even if comparable milk-specific IgE concentrations was revealed in both groups [32].

A weak immunoreactivity was also observed in the range of 10–15 kDa both for pasteurized and baked milk, corresponding to band *g* and *h* in the relative electrophoretic profile illustrated in Figure 1 and putatively attributed to whey proteins (ALA and β-LG). Heat treatments were demonstrated to affect the antigenicity of ALA and β-LG in whey protein isolate. Umesh Kumar Shandilya et al., found that the consumption of sterilized cow milk by WP-sensitized animals caused a significant reduction (*p* ≤ 0.01) of total IgE levels by 43% compared with raw milk WP [43]. Moreover, the aggregation phenomena of β-LG and ALA during heat treatment, could also cause the inhibition of protein uptake by intestinal epithelial cells.

Different results in IgE reactivity were displayed when milk proteins were cooked within a food matrix, such as muffin (Figure 2, Lane 4). In this case, IgE reactivity of milk proteins appeared drastically reduced and only proteins banding between 37 kDa and 20 kDa (Figure 2, Lane 4, corresponding to bands *m*, *n,* and *o* in the respective SDS-PAGE profile) showed a very weak IgE response. As previously described, these bands belong to αS1 casein and β-casein. The scarce IgE reactivity observed for milk proteins baked within a muffin matrix compared to what displayed for baked milk, well highlights the importance of the food matrix in the modulation of the final immunoreactivity of an allergenic food. As already discussed, the occurrence of interaction between proteins and food components could lead to chemical/structural modifications on the protein moiety, with consequent masking of active epitopes and reduction of IgE binding sites. This behavior was already reported in literature. Indeed, in a prospective study, Nowak-Wegrzyn et al. found that heated milk–reactive subjects had significantly larger skin prick test wheals and higher milk-specific and casein-specific IgE levels than other groups. After 3 months of ingesting heated milk products, reactive subjects had significantly smaller skin prick test wheals compared to time 0 and higher casein-IgG4. In conclusion the paper demonstrates that the majority (75%) of children with milk allergy tolerate heated milk [17]. In another study, Kim et al. report that after sequential food challenges with baked cheese and unheated milk in a test population of children previously found tolerant to extensively heated (baked) milk products, approximately 28% and 60% of them were able to tolerate baked milk/baked cheese and unheated milk, respectively with no difference in milk-specific IgE levels between groups [20].

On the other hand, heating milk allergens contained into a food matrix does not completely eliminate the risk of an allergic reaction, because according to the works published, only the reactivity of a few allergens showed to be significantly reduced after the heating applied [20,44]. In this regard, Bloom et al. demonstrated that matrix has an effect on the decrease of IgE immunoreactivity. This was demonstrated by comparing immunoblot analysis of pooled and individual sera of subjects fed with wheat food matrix enriched with milk vs. milk heated under the same conditions. The authors speculated that low reactivity observed in milk containing food matrix was due to the formation of complexes between wheat and milk proteins. However, RBL assay and tests of stimulation of peripheral mononuclear cells obtained from allergic children, showed that there were no differences between heated and unheated proteins, with higher mediator release and higher T-cell stimulation index in tolerant subjects. They hypothesized that the formation of protein complexes during heating could enhance the allergenicity in in vitro systems [32].

### 3.3. In Silico Analyses to Assess IgE Binding Capacity of Milk Products

In the final section of our work we also investigated the immunoreactive epitopes spread along the protein moiety found positive to immunoblot with allergic sera (Figure 2). To this purpose, all peptides obtained from in gel tryptic digestion of protein bands of pasteurized/baked milk and milk-incurred muffin excised from the gel (see Figure 1, Lane 1, Bands *c*–*h*; Lane 4, Bands *m*–*o*) were taken into consideration. The IEDB database was screened to find a match with known milk linear epitopes recognized by *Homo sapiens* as host. The results are summarized in Table 3. In Table S1 of supplementary data, peptide sequences searched in IEDB database along with the epitopic sequences were shown. As for milk (pasteurized and baked) several peptide sequences were found to match with intact epitopes, specifically LGEYGFQNALIVR that was previously attributed to BSA protein (Figure 1, Lane 1, Band *c*) and TPEVDDEALEK, VLVLDTTDYK, and VYVEELKPTPEGDLEILLQK all attributed to β-LG (Figure 1, Lane 1, Band g). Similarly, peptides FFVAPFPEVFGK, EGIHAQQK, and HIQKEDVPSER, obtained from the digestion of selected bands referred to CM incurred muffin sample and assigned to αS1-casein (Figure 1, Lane 4, Band *m*) were found to fully match with immunoreactive epitopes. On the contrary, most of the peptides retrieved by the software were found to overlap with a small portion of the epitope sequence and this was observed both in pasteurized milk and incurred muffin (Table 3, Lane 1 Bands *d*, *e*, *g*, *h* and Lane 4, Bands *m* and *n*). Finally, some peptides belonging to β-LG and identified in band *g* (Figure 1, Lane 1, pasteurized milk) together with those assigned to αS1 casein and β-casein by tryptic digestion of bands *m* and *n* (Figure 1, Lane 4, milk muffin) were found to include short epitopic sequences. Interestingly, two peptides namely YLGYLEQLLR and FFVAPFPEVFGK, identified in bands *m* and *o* (Figure 1, Lane 4) of milk-containing muffin and attributed to αS1 casein, were recognized as immunodominant epitopes since no differences in the epitope specificity between IgG and IgE were highlighted. This directly translates into a higher T cell stimulation capacity than other epitope regions of the αS1 casein [45,46]. In the light of these results, we could conclude that different epitope sequences spread along caseins and whey proteins show to survive to baking at 180 °C (10 min) in heated milk (Figure 2, Lanes 1 and 2). On the contrary, the final immunoreactivity of muffin incurred with milk appeared consistently reduced compared to the not baked food product and the only immunoreactivity observable was ascribable to resistant epitopes belonging to αS1, as demonstrated by proteomic analysis and IEDB search. It is worth to be noted that, even in the aggregated form (MW of band *m* seems slightly higher than the corresponding band *d* of pasteurized milk, see Figure 1), αS1 casein retains its allergenicity.

## 4. Conclusions

In this study, the effects of thermal treatment on cow milk proteins included into a food like muffins, were investigated from a proteomic point of view and for their potential to reduce the allergenic response by immunoblot analysis. To this purpose, pasteurized and baked milk and the inclusion or not into a food was studied in terms of allergenicity retention. The analysis of the protein profile showed that the presence or absence of the food matrix might account for differences detected in the allergen pattern and the resulting immunoreactivity as assessed by SDS-PAGE and immunoblot analysis using a pool of sera of cow’s milk allergic patients.

In the light of our results, milk baked within the muffin matrix might promote formation of complexes with food components inducing a modulation of the immunoreactivity towards milk allergens compared to milk baked in the oven at 180 °C for ten minutes. The interactions between milk proteins and some components of the food matrix during heating seemed to play a role in the possible reduction of allergenicity as assessed by in vitro tests. Further studies employing simulated in vitro gastrointestinal digestion systems will be necessary to better investigate the slight residual IgE reactivity displayed in the CM incurred muffin. Indeed, assessing the fate of allergenic proteins subjected to heat processing techniques in food matrices upon gastrointestinal digestion can help to understand the immunomodulatory effects and the total tolerance of these types of foods in cow milk allergic patients.

## Figures and Tables

**Table 1:** The clinical features of the allergic individuals enrolled in this study.

Serum	Age (Years)	IgE Total (KU/L)	IgE to Cow’s Milk (KU/L)	IgE to Casein (KU/L)	Allergic Reaction Displayed
1	8	5000	62	44	anaphylaxis
2	5	203	100	100	anaphylaxis
3	11	433	54	56	anaphylaxis
4	16	370	87	80	anaphylaxis
5	6	4786	56	34	urthicaria
6	5	4662	100	100	vomit

**Table 2:** Identification of protein bands excised from the SDS gel and analyzed by LC-HR-MS/MS through detection of the proteotypic peptides.

Sample	Band	Accession Number	Allergenic Proteins	Allergen Code	Coverage	Filtered Peptides
Pasteurized Milk/Baked milk	c	A0A140T897	Serum albumin	Bos d 6	38.22	26
	d	P02662	αS1-casein	Bos d 9	33.17	1
		P02663	αS2-casein	Bos d 10	40.54	12
		B5B3R8	αS1-casein	Bos d 9	33.17	1
	e	A0A140T8A9	κ-casein	Bos d 12	23.15	4
		A0A1Y0KDJ6	β-casein	Bos d 11	25	4
		J9UHS4	β-casein	Bos d 11	28.57	1
		P02663	αS2-casein	Bos d 10	22.97	6
	f	B5B0D4	β-lactoglobulin	Bos d 5	30.89	6
		Q28049	α-lactalbumin	Bos d 4	23.,57	4
	g	B5B0D4	β-lactoglobulin	Bos d 5	71.91	12
	h	Q28049	α-lactalbumin	Bos d 4	44.71	5
CM incurred muffins	i	P02662	αS1-casein	Bos d 9	17.28	2
		A0A1Y0KDJ6	β-casein	Bos d 11	9.37	3
	m	B5B3R8	αS1-casein	Bos d 9	39.71	1
		P02662	αS1-casein	Bos d 9	41.58	2
	n	A0A1Y0KDJ6	β-casein	Bos d 11	23.,21	4
	o	P02662	αS1-casein	Bos d 9	8.87	1

**Table 3:** List of potential immunogenic sequences recognized in the peptides identified in specific electrophoretic bands of the milk extracts along with the relevant cow’s milk epitope ID reported in Immune Epitope Database (IEDB).

Protein Band	Peptide Sequence	Epitope ID
c	LGEYGFQNALIVR	235209
d	LHSMK	70444, 115236, 11860, 35531, 56749, 109484, 109828, 115343, 115476
	EDVPSER	663659, 28169, 109358, 30333, 30334, 31120, 31121, 115310, 48707, 78245, 115440, 190571, 68322
	ITVDDK	78138, 115315, 115477, 115479, 115532, 606543
	LNFLK	45706, 78144, 95351, 95560, 115226, 115512
e	EAMAPK	115216
	FFSDK	15893, 30141, 6173, 78257, 115305, 115404, 115449, 115465, 115733, 229682, 229689
	GPFPIIV	115251
g	TPEVDDEALEK	65565, 78111, 96146, 13583, 56146, 95306, 95369, 95922, 96628, 115172, 115519, 146504, 222188
	TKIPAVFK	33732, 95498, 96388, 33733, 46987, 78279, 96064, 96968, 115173, 115185, 115313, 222020
	VLVLDTDYKK	96517, 69827, 95545, 95579, 222193, 223163
	IPAVFK	33732, 96388, 31382, 33733, 46987, 78279, 96064, 96968, 98849, 115173, 115185, 115313, 222020
	VLVLDTDYK	96091, 96517, 69827, 95545, 95579, 222193, 223163
	LSFNPTQLEEQCHI	39349, 2820, 115382, 24090, 95389, 95574, 98777, 98893, 115427
	VYVEELKPTPEGDLEILLQK	72178, 32907, 96569, 97098, 32908, 72177, 95347, 96219, 98752, 98760, 99028, 99036, 224315
h	EQLTK	115234, 227758, 558421
m	FFVAPFPEVFGK	38207, 43705, 15930, 15931, 44794, 67707, 69660, 110049, 115396, 115467, 190478, 659427, 659428
	HQGLPQEVLNENLLR	115282, 31145, 50721, 50900, 109844, 115311, 675165
	EPMIGVNQELAYFYPELFR	12961, 13714, 13715, 13716, 23078, 45538, 45539
	YLGYLEQLLR	74689, 30334, 74687, 74688, 115482, 14100, 109358, 110060, 115060, 115122, 115213, 115440, 190580, 229693
	VNELSK	68473, 115253, 12896, 20548, 68472, 70058, 70059, 78158, 108948, 110049, 115068, 115531, 115544, 190572, 229694
	EGIHAQQK	24814, 12187, 30400, 41811, 115236, 24813, 109484, 109828, 115306, 115476, 190445, 606414
	HIQKEDVPSER	68322, 78245, 28169, 31120, 31121, 48707, 109358, 115310, 190571, 663657, 663658, 663659
	EDVPSER	68322, 30334, 78245, 28169, 30333, 31120, 31121, 48707, 109358, 115310, 115440, 190571, 663659
n	GPFPIIV	115251, 658276, 670213, 671639, 673180, 673307, 688313
	DMPIQAFLLYQEPVLGPVR	42283, 75481, 115675, 115796, 115847, 115866
	AVPYPQR	51169, 70443, 115430, 115439, 115495, 115694, 657013, 657014
	VLPVPQK	52358, 52359, 115495, 115835, 161678, 227654, 679766, 735655
o	FFVAPFPEVFGK	15930, 15931, 659427, 659428, 38207, 190478, 43705, 44794, 115396, 115467, 67707, 69660, 110049

## Supplementary Materials

The following are available online at https://www.mdpi.com/2072-6643/11/7/1536/s1.
