# Supplementary material for: Modulation of Milk Allergenicity by Baking Milk in Foods: A Proteomic Investigation
Source: Nutrients. 2019 Jul 6;11(7):1536. doi: 10.3390/nu11071536 (PMC6683078; doi:10.3390/nu11071536)
Supplement: Supplementary file 1 [file nutrients-11-01536-s001.pdf]

Table S1

Protein Band	Peptide sequence	Epitope ID	Epitopic sequence
**c**	LGEYGFQNALIVR	235209	**LGEYGFQNALIVR**
**d**	LHSMK	70444	VPQLEIVPNSAEER**LHSMK**E
		115236	ER**LHSMK**EGIHAQQKEPMIG
		11860	EER**LHSMK**EG
		35531	LEIVPNSAEER**LHSMK**EG + PHOS(S7)
		56749	SAEER**LHSMK**
		109484	LKKYKVPQLEIVPNSAEER**LHSMK**EGIHAQQKE
		109828	R**LHSMK**EGIHAQQKEPMIGVNQELAYFYPE
		115343	LEIVPNSAEER**LHSMK**EGIH
		115476	SAEER**LHSMK**EGIHAQQKEP
	EDVPSER	663659	HIQK**EDVPSER**
		28169	IQK**EDVPSER**
		109358	ISSSEEIVPNSVEQKHIQK**EDVPSER**YLGYLEQLLR
		30333	K**EDVPSER**YL
		30334	K**EDVPSER**YLGYLEQLLRLK
		31120	KHIQK**EDVPSER**
		31121	KHIQK**EDVPSER**YLGYLE
		115310	KHIQK**EDVPSER**YLGYLEQL
		48707	PNSVEQKHIQK**EDVPSER** + PHOS(S3)
		78245	PNSVEQKHIQK**EDVPSER**YL
		115440	Q**KEDVPSER**YLGYLEQLLRL
		190571	VEQKHIQK**EDVPSER**
		68322	VEQKHIQK**EDVPSER**YLGYL
	ITVDDK	78138	EEVK**ITVDDK**HYQKALNEIN
		115315	K**ITVDDK**HYQKALNEINQFY
		115477	SAEVATEEVK**ITVDDK**HYQK
		115479	SEESAEVATEEVK**ITVDDK**H
		115532	VATEEVK**ITVDDK**HYQKALN
		606543	VK**ITVDDK**HYQKALNEIN
	LNFLK	45706	NR**LNFLK**KIS
		78144	EKNR**LNFLK**KISQRYQKFALPQYLKT
		95351	EKNR**LNFLK**K
		95560	**LNFLK**KISQR
		115226	EKNR**LNFLK**KISQRYQKFAL
		115512	TKKTKLTEEEKNR**LNFLK**KI
**e**	EAMAPK	115216	**EAMAPK**HKEMPFPKYPVEPF
	FFSDK	15893	**FFSDK**IAKYI
		30141	KDER**FFSDK**I
		6173	CEKDER**FFSDK**IAK
		78257	R**FFSDK**IAKYIPIQYVLSRY
		115305	KDER**FFSDK**IAKYIPIQYVL
		115404	NQEQPIRCEKDER**FFSDK**IA
		115449	QPIRCEKDER**FFSDK**IAKYI
		115465	RCEKDER**FFSDK**IAKYIPIQ
		115733	ER**FFSDK**IAK
		229682	IHCEKDER**FFSDK**IAKYI
		229689	IRCEKDER**FFSDK**IAKYI
	GPFPIIV	115251	FLLYQEPVLGPVR**GPFPIIV**
**g**	TPEVDDEALEK	65565	**TPEVDDEALEK**
		78111	CLVR**TPEVDDEALEK**FDKAL
		96146	CLVR**TPEVDDEALEK**
		13583	ENSAEPEQSLVCQCLVR**TPEVDDEALEK**FDKALKALPM
		56146	R**TPEVDDEALEK**FDKALKAL
		95306	**DDEALE**
		95369	**EVDDEA**
		95922	**TPEVDD**
		96628	LVR**TPEVDDEALEK**F

	115172	ACQCLVR**TPEVDDEALEK**FD
	115519	**TPEVDDEALE**
	146504	QCLVR**TPEVDDEALEK**
	222188	CLVR**TPEVDDEALEK**FDK
TKIPAVFK	33732	K**TKIPAVFK**I
	95498	**KIPAVF**
	96388	IIAEK**TKIPAVFK**ID
	33733	K**TKIPAVFK**IDA
	46987	**PAVFK**
	78279	**TKIPAVFK**IDALNENKVLVL
	96064	AEK**TKIPAVFK**IDAL
	96968	**TKIPAVFK**IDALNEN
	115173	AEK**TKIPAVFK**IDALNENKV
	115185	AQKKIIAEK**TKIPAVF**KIDA
	115313	KIIAEK**TKIPAVFK**IDALNE
	222020	AEK**TKIPAVFK**IDALNEN
VLVLDTDYKK	96517	K**VLVLDTDYKK**YLLF
	69827	**VLVLDTDYK**
	95545	**LDTDYK**
	95579	**LVLDTD**
	222193	DALNENK**VLVLDTDYKK**Y
	223163	K**VLVLDTDYKK**YLLFCME
IPAVFK	33732	KTK**IPAVFK**I
	96388	IIAEKTK**IPAVFK**ID
	31382	K**IPAVFK**IDALNENKVLVLDT
	33733	KTK**IPAVFK**IDA
	46987	**PAVFK**
	78279	TK**IPAVFK**IDALNENKVLVL
	96064	AEKTK**IPAVF**KIDAL
	96968	TK**IPAVFK**IDALNEN
	98849	K**IPAVFK**IDA
	115173	AEKTK**IPAVFK**IDALNENKV
	115185	AQKKIIAEKTK**IPAVFK**IDA
	115313	KIIAEKTK**IPAVFK**IDALNE
	222020	AEKTK**IPAVFK**IDALNEN
VLVLDTDYK	96091	ALNENK**VLVLDTDYK**
	96517	K**VLVLDTDYK**KYLLF
	69827	**VLVLDTDYK**
	95545	**LDTDYK**
	95579	**LVLDTD**
	222193	DALNENK**VLVLDTDYK**KY
	223163	K**VLVLDTDYK**KYLLFCME
LSFNPTQLEEQCHI	39349	**LSFNPTQLEEQCHI**
	2820	ALPMHIR**LSFNPTQLEEQCHI**
	115382	MHIR**LSFNPTQLEEQCHI**
	24090	HIR**LSFNPTQLEEQCHI**
	95389	**FNPTQLEEQCHI**
	95574	**LSFNPT**
	98777	**FNPTQLEEQC**
	98893	**LSFNPTQLEE**
	115427	**PTQLEEQCHI**
VYVEELKPTPEGDLEILLQK	72178	**VYVEELKPTPEGDLEILLQK**
	32907	**KPTPEGDLEI**
	96569	**LKPTPEGDLEILLQK**
	97098	**VYVEELKPTPEGDLE**
	32908	**KPTPEGDLEILLQK**
	72177	**VYVEELKPTP**
	95347	**EILLQK**
	96219	**EELKPTPEGDLEILL**
	98752	**EGDLEILLQK**
	98760	**ELKPTPEGDL**
	99028	**TPEGDLEILL**

		99036	**VEELKPTPEG**
		224315	**VEELKPTPEGDLEILLQK**
**h**	EQLTK	115234	**EQLTK**CEVFR
		227758	**EQLTK**CEVFRELKDLKGY
		558421	**EQLTK**CEVFRELKDL
**m**	FFVAPFPEVFGK	38207	LNENLLR**FFVAPFPEVFGK**E
		43705	NENLLR**FFVAPFPEVFGK**EK
		15930	**FFVAPFPEVF**
		15931	**FFVAPFPEVFGK**EK
		44794	NLLR**FFVAPFPEVFGK**EK
		67707	**VAPFPEVFGK**
		69660	VLNENLLR**FFVAPFPEVFGK**EK
		110049	VLNENLLR**FFVAPFPEVFGK**EKVNELSKDIGS
		115396	NLLR**FFVAPFPEVFGK**EKVN
		115467	R**FFVAPFPEVFGK**EKVNELS
		190478	LR**FFVAPFPEVFGK**E
		659427	**FVAPFPEVF**
		659428	**FVAPFPEVFGK**
	HQGLPQEVLNENLLR	115282	HPIK**HQGLPQEVLNENLLR**F
		31145	K**HQGLPQEVLNENLLR**FF
		50721	**QEVLNENLLR**
		50900	**QGLPQEVLNE**
		109844	RPKHPIK**HQGLPQEVLNENLLR**FFVAPFPEV
		115311	K**HQGLPQEVLNENLLR**FFVA
		675165	RPKHPIK**HQGLPQEVLNENLLR**F
	EPMIGVNQELAYFYPELFR	12961	**ELAYFYPELF**
		13714	**EPMIGVNQEL**
		13715	**EPMIGVNQELAY**
		13716	**EPMIGVNQELAYFYPELF**
		23078	**GVNQELAYFY**
		45538	**NQELAYFYPE**
		45539	**NQELAYFYPELF**
	YLGYLEQLLR	74689	**YLGYLEQLLR**LKKYKVPQLE
		30334	KEDVPSER**YLGYLEQLLR**LK
		74687	**YLGYLEQLLR**
		74688	**YLGYLEQLLR**LKKYKVPQ
		115482	SER**YLGYLEQLLR**LKKYKVP
		14100	ER**YLGYLEQLLR**LK
		109358	ISSSEEIVPNSVEQKHIQKEDVPSER**YLGYLEQLLR**
		110060	VPSER**YLGYLEQLLR**LKKYKVPQLEIVPNS
		115060	G**YLEQL**
		115122	VPSER**YLGYLEQLLR**LKK
		115213	DVPSER**YLGYLEQLLR**LKKY
		115440	QKEDVPSER**YLGYLEQLLR**L
		190580	VPSER**YLGYLEQLLR**
		229693	KDDVPSER**YLGYLEQLLR**LK
	VNELSK	68473	VFGKEK**VNELSK**DIGSESTE
		115253	FPEVFGKEK**VNELSK**DIGSE
		12896	EK**VNELSK**DI
		20548	GKEK**VNELSK**
		68472	VFGKEK**VNELSK**DIGSES + PHOS(S16, S18)
		70058	**VNELSK**DIGS
		70059	**VNELSK**DIGSESTEDQAM + PHOS(S10, S12)
		78158	FPEVFGKEK**VNELSK**DIGSESTE
		108948	FGKEK**VNELSK**DIGSESTEDQAMEDIKQMEAES
		110049	VLNENLLRFFVAPFPEVFGKEK**VNELSK**DIGS
		115068	KEK**VNELSK**DIG
		115531	VAPFPEVFGKEK**VNELSK**DI
		115544	**VNELSK**DIGSESTEDQAMED
		190572	VFGKEK**VNELSK**DIG
		229694	KEK**VNELSK**DIGSES
	EGIHAQQK	24814	HSMK**EGIHAQQK**EPMIGVNQ

		12187	**EGIHAQQK**EP
		30400	K**EGIHAQQK**EPM
		41811	MK**EGIHAQQK**
		115236	ERLHSMK**EGIHAQQK**EPMIG
		24813	HSMK**EGIHAQQK**EPMIGV
		109484	LKKYKVPQLEIVPNSAEERLHSMK**EGIHAQQK**E
		109828	RLHSMK**EGIHAQQK**EPMIGVNQELAYFYPE
		115306	K**EGIHAQQK**EPMIGVNQELA
		115476	SAEERLHSMK**EGIHAQQK**EP
		190445	HSMK**EGIHAQQK**EPM
		606414	**EGIHAQQK**
	HIQKEDVPSER	68322	VEQK**HIQKEDVPSER**YLGYL
		78245	PNSVEQK**HIQKEDVPSER**YL
		28169	**IQKEDVPSER**
		31120	K**HIQKEDVPSER**
		31121	K**HIQKEDVPSER**YLGYLE
		48707	PNSVEQK**HIQKEDVPSER** + PHOS(S3)
		109358	ISSSEEIVPNSVEQK**HIQKEDVPSER**YLGYLEQLLR
		115310	K**HIQKEDVPSER**YLGYLEQL
		190571	VEQK**HIQKEDVPSER**
		663657	**HIQKEDVPS**
		663658	**HIQKEDVPSE**
		663659	**HIQKEDVPSER**
	EDVPSER	68322	VEQKHIQK**EDVPSER**YLGYL
		30334	K**EDVPSER**YLGYLEQLLRLK
		78245	PNSVEQKHIQK**EDVPSER**YL
		28169	IQK**EDVPSER**
		30333	K**EDVPSER**YL
		31120	KHIQK**EDVPSER**
		31121	KHIQK**EDVPSER**YLGYLE
		48707	PNSVEQKHIQK**EDVPSER** + PHOS(S3)
		109358	ISSSEEIVPNSVEQKHIQK**EDVPSER**YLGYLEQLLR
		115310	KHIQK**EDVPSER**YLGYLEQL
		115440	QK**EDVPSER**YLGYLEQLLRL
		190571	VEQKHIQK**EDVPSER**
		663659	HIQK**EDVPSER**
**n**	GPFPIIV	115251	FLLYQEPVLGPVR**GPFPIIV**
		658276	EPVLGPVR**GPFPIIV**
		670213	LLYQEPVLGPVR**GPFPIIV**
		671639	LYQEPVLGPVR**GPFPIIV**
		673180	PVLGPVR**GPFPIIV**
		673307	QEPVLGPVR**GPFPIIV**
		688313	YQEPVLGPVR**GPFPIIV**
	DMPIQAFLLYQEPVLGPVR	42283	**MPIQAFLLYQEP**
		75481	**YQEPVLGPVR**
		115675	**AFLLYQEPVL**
		115796	**IQAFLLYQEP**
		115847	**LLYQEPVLGP**
		115866	**MPIQAFLLYQ**
	AVPYPQR	51169	QK**AVPYPQR**D
		70443	VPQK**AVPYPQR**D
		115430	PVPQK**AVPYPQR**DMPIQAFL
		115439	QK**AVPYPQR**DMPIQAFLLYQ
		115495	SQSKVLPVPQK**AVPYPQ**RDM
		115694	**AVPYPQR**DMP
		657013	**AVPYPQR**DMPIQA
		657014	**AVPYPQR**DMPIQAF
	VLPVPQK	52358	QSK**VLPVPQK**
		52359	QSK**VLPVPQK**AV
		115495	SQSK**VLPVPQK**AVPYPQRDM
		115835	K**VLPVPQK**AV
		161678	QSK**VLPVPQK**AVP

		227654	VEKLHLPLPLVQSWMHQPPQPLSPTVMFPPQSVLSLSQPK**VLPVPQK**AV
		679766	SLSQSK**VLPVPQK**AVPYPQ
		735655	**VLPVPQ**
**o**	FFVAPFPEVFGK	15930	**FFVAPFPEVF**
		15931	**FFVAPFPEVFGKEK**
		659427	**FVAPFPEVF**
		659428	**FVAPFPEVFGK**
		38207	LNENLLR**FFVAPFPEVFGK**E
		190478	LR**FFVAPFPEVFGK**E
		43705	NENLLR**FFVAPFPEVFGK**EK
		44794	NLLR**FFVAPFPEVFGK**EK
		115396	NLLR**FFVAPFPEVFGKEK**VN
		115467	R**FFVAPFPEVFGK**EKVNELS
		67707	**VAPFPEVFGK**
		69660	VLNENLLR**FFVAPFPEVFGK**EK
		110049	VLNENLLR**FFVAPFPEVFGK**EKVNELSKDIGS
